# Targeting dipeptidyl peptidase-8/9 to combat inflammation-induced osteoclastogenesis in RAW264.7 macrophages and analysis of anti-osteoclastogenesis potential of chrysin

**DOI:** 10.22038/ijbms.2025.82219.17784

**Published:** 2025

**Authors:** Syed Sufian Ahmad, Faraha Ahmed, Sayeed Ahmad, Anuja Krishnan, Mohammad Ahmed Khan

**Affiliations:** 1 Department of Pharmacology, School of Pharmaceutical Education and Research, Jamia Hamdard, Hamdard, New Delhi – 110062, India; 2 Bioactive Natural Product Laboratory, School of Pharmaceutical Education and Research, Jamia Hamdard, Hamdard, New Delhi – 110062, India; 3 Department of Molecular Medicine, School of Interdisciplinary Science and Technology, Jamia Hamdard, Hamdard, New Delhi – 110062, India

**Keywords:** Chrysin, Dipeptidyl peptidase 8, Dipeptidyl peptidase 9, Inflammatory – osteoclastogenesis, M1 macrophage, RAW264.7 cells

## Abstract

**Objective(s):**

Osteoclasts drive bone resorption under inflammation, with cytokines promoting osteoclastogenesis. The role of proline enzymes like dipeptidyl peptidase-8 and 9 (DPP-8/9) in this process remains unclear. This study aimed to explore the DPP-8/9 involvement in inflammation-driven osteoclastogenesis using the RAW264.7 macrophage model.

**Materials and Methods:**

Receptor activator of nuclear factor-κB ligand (RANKL) and lipopolysaccharide (LPS) induced osteoclastogenesis, raising interleukin-6 (IL-6), tumor necrosis factor (TNF-α), and IL-23 levels. Using RAW264.7 cells, DPP-8/9 protein and tartrate-resistant acid phosphatase (TRAPc) were assayed. Antibodies for cluster of differentiation (CD86 and CD206) were used to analyze macrophage polarization, while molecular docking was used to assess flavonoid binding to DPP-8/9. Western blot confirmed DPP-8/9 expression in treated macrophages.

**Results:**

Administering RANKL and LPS increased IL-6 and TNF-α levels, significantly promoting osteoclastogenesis in RAW264.7 macrophages. This treatment also elevated the levels of the inflammatory macrophage marker IL-23. Osteoclast formation was confirmed by measuring TRAPc levels in the culture. Analysis of the cell supernatant revealed elevated DPP-8/9 levels in the RANKL+LPS group. Inhibition of DPP-8/9 with 1G244 decreased inflammatory cytokines and TRAPc levels in the cell culture. Molecular docking analysis of various flavonoids identified chrysin as a potential molecule with sufficient binding energy against DPP-8/9, a finding confirmed by blotting assay.

**Conclusion:**

This study emphasizes the involvement of DPP-8/9 in inflammatory osteoclastogenesis in RAW264.7 macrophages. Inhibition of DPP-8/9 reduced osteoclastogenesis markers and inflammatory cytokines levels, indicating decreased osteoclast formation. Additionally, chrysin demonstrated potential as an anti-DPP-8/9 agent, highlighting its possible role in future therapeutic strategies targeting inflammation-induced osteoclastogenesis.

## Introduction

The immune system is an intricate nexus of cells and tissues working closely with other body parts to keep us healthy (1). In vertebrate evolution, the simultaneous emergence of immune cells and bone cells confirms that our evolutionary development was not a random occurrence (2). It is crucial to recognize the interdependence of the osteo-immune system whenever studying bone or immune function, as these systems do not operate in isolation but are deeply intertwined and constantly influence each other (3). A meta-analysis encompassing 70 studies and a sample of 800,457 women revealed a global osteoporosis prevalence of 23.1% among women and 11.7% among men (4). By 2050, fractures due to osteoporosis are expected to surge significantly in men (310%) and women (240%)—highlighting a major drift in the global burden of the disease (5). Studies suggest immune cells are essential for bone health, particularly in initiating bone resorption (3). Pioneering research from the 1970s shed light on the involvement of crucial factors derived from immune cells in the formation of bone-resorbing osteoclasts, giving rise to the field of osteoimmunology (6, 7). This concept of mutual dependency of two systems will help explore the new molecular targets and pathways, highlighting this complex yet crucial connection. It might help design novel approaches to managing various inflammation-induced bone pathologies. 

Inflammation, orchestrated by our immune system in response to various triggers, is a critical regulator in maintaining tissue balance. However, when inflammation becomes dysregulated, it significantly threatens bone health, leading to conditions such as osteoporosis (8). Within this context, macrophages, key players in the immune response, assume a central role in bone metabolism (9,10). Macrophages can adopt different roles depending on their surrounding environment. Proinflammatory cytokines activate M1-like macrophages and play a key role in driving inflammation (11). In contrast, M2-like macrophages are involved in resolving inflammation and clearing out dead cells through efferocytosis, helping restore tissue balance after an inflammatory response (11, 12). Their capacity to transform into osteoclasts and regulate bone resorption through the production of proinflammatory agents, such as tumor necrosis factor-α (TNF-α), interleukin-23 (IL-23), and interleukin-6 (IL-6), emphasize their importance in maintaining skeletal equilibrium (13). These inflammatory cytokines facilitate the differentiation of macrophages into mature osteoclasts, as indicated by elevated levels of tartrate-resistant acid phosphatase (TRAPc) (14, 15). Despite the formidable efficiency of osteoclasts in bone resorption, they present prime targets for addressing osteoporosis (10, 16). However, conventional anti-resorptive therapies like bisphosphonates face obstacles due to adverse effects such as jaw necrosis (osteonecrosis of the jaw (ONJ)), arrhythmia (atrial fibrillation), and kidney failure associated with their long-term use (17, 18). Additionally, osteoclasts develop resistance to apoptosis in inflammatory settings, leading to treatment failure (19). It highlights the urgent need for alternative therapeutic strategies that effectively target osteoclasts to counter excessive bone loss. One promising approach is to disrupt osteoclast formation at its earliest stage by targeting precursor cells in inflammatory environments before they mature and contribute to bone degradation. 

Recent advancements in understanding cellular proteases have unveiled the diverse roles of proline-specific serine proteases dipeptidyl peptidases 8 and 9 (DPP-8/9) beyond simple protein degradation, rendering them promising targets for therapeutic interventions (20, 21). DPP-8/9 members of the proline-specific serine DPP family, have attracted attention due to their structural similarities to DPP-4 and their prevalence in inflammation-prone regions (22, 23). The serine family proteases DPP-8/9 are proline-specific. They share remarkable homological, biochemical, and substrate-specific similarities. DPP-8/9 are highly found in macrophages and monocyte regions and exhibit over threefold increased expression during the activation of M1 inflammatory macrophages (24, 25). Research indicates that DPP-8/9 inhibition can effectively decline macrophage activation, significantly reducing inflammatory cytokines (IL-6 and TNF-α) (26). *In vitro *experiments showed that inhibition of DPP-8/9 in THP-1 cells significantly decreased the transformation of macrophages into inflammatory macrophages (27). The inhibition of DPP-8/9 has shown potential in dampening M1 macrophage activation and suppressing proinflammatory responses, suggesting their therapeutic promise in inflammatory conditions (20,24). The widespread expression of DPP-8/9 across various tissues and cell lines underlines their potential as therapeutic targets for inflammatory diseases (20). While their involvement in inflammatory diseases like colitis, asthma, and atherosclerosis is well-documented, their functions in bone biology remain elusive (28–30). The anti-inflammatory effect demonstrated by DPP-8/9 inhibition suggests its potential as a therapeutic target. This highlights the necessity to thoroughly investigate their involvement in inflammation-driven osteoclast formation in RAW264.7 macrophages. There are no well-established inhibitors for DPP-8/9, apart from 1G244, which has shown undesirable effects in both *in vitro *and *in vivo *studies (27,31). The persisting concern around the narrow therapeutic window of DPP-8/9 inhibitors, including 1G244, necessitates further exploration of molecules with better safety and efficacy.

 Thus, this study aims to elucidate the effects of selective DPP-8/9 inhibition on RAW-osteoclast (osteoclasts transformed from RAW264.7 macrophages) formation using an *in vitro *model of inflammation-induced osteoclastogenesis. Additionally, this study aims to evaluate flavonoids as DPP-8/9 inhibitors using computational molecular docking, which helps identify potential compounds that effectively target the DPP-8/9 protein. The binding energy between the protein and ligands serves as a key indicator of the specificity and stability of these interactions. Therefore, molecular docking becomes crucial for identifying ligand-protein binding interactions. The flavonoids selected for the docking study have exceptional anti-oxidant properties and notable bone health benefits, making them highly suitable candidates aligned with the goals of this research (32). Among all the docked flavonoids, chrysin, flavone class flavonoid demonstrated the highest binding energy, which means better affinity and stability for DPP-8/9 proteins. Moreover, chrysin’s reported specificity for serine endopeptidases highlights its potential as a leading compound against DPP-8/9, making it a promising candidate for *in vitro *investigations (33). This research is the first to investigate the impact of DPP-8/9 inhibition on the transformation of macrophages into osteoclasts in RAW264.7 cell lines. Through comprehensive analyses, this study emphasizes the potential of these proteases as medicinal for addressing inflammatory bone loss. By offering new perspectives on the involvement of DPP-8/9 in inflammation and osteoclastogenesis, it expands our understanding of their role in bone metabolism. Furthermore, the findings support the pursuit of developing highly specific inhibitors for DPP-8/9.

## Materials and Methods

### Drugs and reagents

Chrysin (5,7-dihydroxyflavone, >97% purity) (cat no: 95082) and 1G244 (2-Amino-4-{4-[bis(4-fluorophenyl) methyl] piperazin-1-yl}-1-(2,3-dihydro-1H-isoindol-2-yl) butane-1,4-dione) (cat no: 847928-32-9) were purchased from Sigma Aldrich Chemical Co. Inc., (Milwaukee, WI, USA). Dulbecco’s modified Eagle’s medium (DMEM) (cat no: 12800082), fetal bovine serum (FBS) (cat no: 10082147), phosphate buffer saline (PBS) (cat. no: 10082147), and lipopolysaccharide (LPS) (cat no: L23353) were purchased from Thermo Fisher, MA, USA. Antibiotics like penicillin (100 U/ml) + streptomycin (100 ug/ml) (cat no: BMC1030) and other culture reagents were purchased from Abbkine Scientific Co. Ltd., USA. ELISA kits for tartrate-resistant acid phosphatase (TRAcP) (cat no. EM1424), TNF-α (cat. no. EM0183), IL-23 (cat. no: EM0114), and IL-6 (cat. no: EM0121) were purchased from Fine Test, Wuhan Fine Biotech Co. Ltd, Wuhan, Hubei, China. All the chemicals and reagents used in this study were of analytical grade. 

### Molecular docking analysis

The receptor’s binding affinity was determined through a scoring function evaluating ligands with minimal interaction. AutoDock 4.2 was employed for computational investigations, utilizing 3D structures obtained from PDB ID for selected proteins DPP-8 (6EOT) and DPP-9 (6EOR) (34). Ligand structures, sourced from PubChem, underwent preparation with Ligprep using OPLS3e as the force field. AutoGrid in AutoDock 4.2 was used to generate grid maps, composed of 92×92×92 grid points in x, y, and z dimensions, respectively, with a 0.375 Å spacing to encompass the entire monomer (35). All the conformations of different molecules were grouped into clusters with a root mean square deviation (RMSD) of less than 2.0 Å. The two representative structures were chosen from the top two clusters. Subsequently, molecular docking was performed with prepared ligands and the selected receptor grid, and results were generated and exported. 

### Culture of osteoclasts from RAW264.7 macrophages

RAW 264.7 murine macrophages (ATCC no: TIB-71) were obtained from the National Centre for Cell Sciences (NCCS), Pune, India. The RAW 264.7 cells were cultured in Dulbecco’s Modified Eagle’s Medium (DMEM) supplemented with 10% heat-inactivated fetal bovine serum (FBS), 100 µg/ml of penicillin, and 100 µg/ml of streptomycin. The cultures were maintained at 37 °C in a 5% CO_2_ humidified incubator. For the induction of osteoclastogenesis, the specified number of cells (5 × 10^5^/well) were seeded in a 96-well tissue culture plate with receptor activator of nuclear factor kappa B ligand (RANKL) at a concentration of 50 ng/ml for seven days, following established protocols (36). The differentiation of RAW 264.7 macrophages into multinucleated osteoclasts was monitored *in vitro *over ten days, with day 0 marked as the day when RANKL was added. The culture media were replaced every two days to maintain signaling cues. After 7 days of RANKL administration (except in the normal grp), all the cells were divided into multiple groups.

### Cell viability assay

Cell survival analysis was assessed with the modified MTT (3-[4,5-dimethylthiazol-2-yl]-2,5 diphenyl tetrazolium bromide) colorimetric assay. For the 1G244 sample cytotoxicity assay, the manufacturer’s protocol (EZcount^TM^ HIMEDIA-MTT Cell Assay kit) was followed (37). Cells were seeded at a density of 5 x 106 cells in a 150 µl volume per well of a 96-well plate and allowed to grow for two days in the presence and absence of RANKL and LPS at 37 °C with 5% CO_2_ for 48 hr. Modified tetrazolium dye (20 µl) was added to individual wells, and absorbances were read at 570 nm after overnight incubation with the dye. All measurements were taken in triplicate, and the percentage of cell viability was determined using the appropriate equation.

### Flow cytometry for macrophage polarization assessment

The polarization of RAW264.7 cells was evaluated after treatment with LPS + RANKL by analyzing the expression of specific surface markers through flow cytometry. CD86 was used to quantify the proinflammatory M1 macrophage population, and CD206 was used to quantify the anti-inflammatory M2-like macrophage population, with PE Rat Anti-mouse CD86 and 647 Rat Anti-mouse CD206 antibodies (BD BioScience, USA). M1 macrophages were identified as CD86+ (CD206-) cells, while M2 macrophages were identified as CD206+ (CD86-) cells (38). For the experiment, cells were seeded in 24-well plates at 50000 cells/well, and LPS (20 ng/ml) and RANKL (50 ng/ml) were used to trigger the inflammation in the cell. Cells were either pretreated with or without 1G244 (10 and 20 µM) and incubated for 48 hr. Following incubation, cells were harvested by trypsinization (250 µl of Trypsin in EDTA) and collected into separate FACS flow tubes. The cell pellets were washed twice with 1 ml of PBS-FBS 1%, followed by centrifugation at 1500 rpm for 10–15 min at 37 °C. The supernatant was carefully removed, and any remaining buffer was eliminated. Cells were resuspended in a fixation solution (Thermo Fisher, UK) for 25 min at 4 °C. Following centrifugation, the cells were washed twice and supplemented with FBS and saponin (Thermo Fisher, UK). The pellets were resuspended in 1.50 µl of PE Rat Anti-mouse CD86 and Alexa Fluor® labeled 647 Rat Anti-mouse CD206 antibodies (BD BioScience). The cells were incubated on ice for 20-30 min, away from light. After two washes with PBS-FBS, the cells were resuspended in a Cell-Fix solution (300 µl) (Thermo Fisher, UK). The tubes were then analyzed using FACSCanto™ Flow Cytometer (BD Biosciences), and cell populations were sorted and analyzed using QuestPro software (version 2.0). To maintain result accuracy, all samples were analyzed within three hours to prevent fluorescence alterations that might affect the experimental findings.

### TRAPc staining of RAW264.7 cells

RAW264.7 cells were maintained for five days in the presence of RANKL (50 ng/ml) following established protocols, then fixed using 4% paraformaldehyde in PBS at 4 °C for 60 min at 20–25 °C. The cells were treated with 0.2% Triton X-100 at 25 °C for 5 min and rinsed with PBS. For TRAP staining, cells were incubated with a solution of 0.01% naphthol (Sigma-Aldrich), 0.05% fast red violet salt, 50 mM sodium tartrate, and 90 mM sodium acetate (pH 5.0), then rinsed with PBS.

### Estimation of TNF-α, IL-23 and IL-6 levels

To estimate the cytokines, the RAW264.7 cells were maintained at an initial density of 8000 cells/well in a 96-well plate treated with RANKL and LPS to stimulate the inflammatory cytokines secretions in the culture. Then, the cells were treated with and without selective DPP-8/9 inhibitor, 1G244 (10 & 20 μM), and placed in the humidified 5% CO_2_ incubator for 72 hr. After treatment, cells were aspirated using phosphate buffer saline (PBS) (39, 40). Further, it was centrifuged for 5 min at 4000 rpm to obtain a clear supernatant. The supernatant was carefully collected by pipetting the medium from each well into labeled microcentrifuge tubes. The collected supernatants are then centrifuged at 1,000-2,000x g for 10–12 min to clear any cell debris. Using commercially available ELISA kits, the clarified supernatants are shifted to fresh tubes (stored at -80 °C if not used immediately) to estimate Interleukin-6 levels, Interleukin-23 levels, and TNF-α levels.

### DPP enzyme assay

To assess the inhibitory impact of drugs on the DPP-8/9 protein, the cell lysate from RAW264.7 cells treated with RANKL (50 ng/ml) was utilized for the assay following the administration of enzyme inhibitors. The assay was conducted in 96-well plates with a final volume of 100 µl for each well. Within the total volume of 100 µl, 1G244 was present at a concentration of 10 µM. The substrate, H-Ala-Pro-pNA, in each enzyme assay had a concentration of 1 mM, and 0.1% DMSO served as a solvent. After the enzyme cleaved the substrate, the released pNA induced a colorimetric change detectable by absorbance at 405 nm (26, 31). Absorbance measurements were taken at 37 °C to monitor enzymatic activity. The difference in absorbance was calculated by subtracting the initial absorption reading at 0 min (t_0_) from the final absorption reading at 60 min (t_60_). Subsequently, the change in absorption between the two time points was divided by the assay duration (60 min). The percentage of DPP-8/9 activity was determined using the following equation: ΔOD drug / ΔOD control × 100%.

### Western blotting

For western blotting, cells were lysed with the help of Tris buffer (50 mM, pH 7.6) with Triton X-100 (0.1%), NaCl (150 mM), and a complete protease inhibitor cocktail (Sigma Aldrich Chemical Co. Inc., Milwaukee, USA), dissolved according to the instructions. Cell lysates underwent separation via sodium dodecyl sulfate polyacrylamide gel electrophoresis (SDS-PAGE) (12%) using the Laemmli buffer system and were subsequently transferred to a nitrocellulose membrane through electroblotting. The membranes were blocked for one hour at room temperature with non-fat dried milk (5% w/v) and then incubated overnight at 4 °C with primary antibodies (1:1000 DPP-8 and 1:1000 DPP-9 from Affinity Biosciences, USA). The membranes were incubated for two hours with secondary HRP-conjugated antibodies (Abbkine Scientific Co. Ltd, USA) at room temperature (27,31). Bands were developed using ECL Prime reagents (Sigma Aldrich Chemical Co. Inc., Milwaukee, WI, USA). Rainbow markers (Sigma Aldrich Chemical Co. Inc., Milwaukee, WI, USA) were utilized for molecular weight determinations. Protein pattern images were captured using an Image Quant LAS 500 scanner (GE Healthcare, USA).

### Statistical analysis

All data are expressed as mean ± SEM, and P-value≤0.05 was considered significant. Statistical analysis was carried out using Graph Pad Prism Version 9.5.3.733 (Graph Pad Software, California, USA). The parameters were analyzed through one-way ANOVA followed by Tukey-Kramer’s *post hoc* test. 

## Results

### Effect of 1G244 on RAW246.7 cell viability

**Figure 1:**
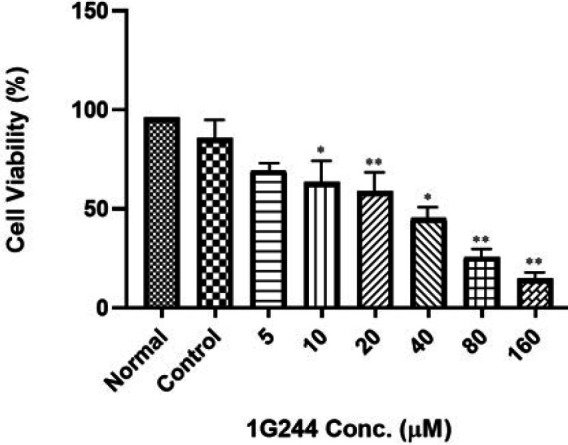
1G244 effect on RAW264.7 cells viability

Cell viability was determined using the MTT assay to investigate the effect of 1G244 on RAW264.7 cells over 48 hr. This assay aimed to exclude the possibility that the inhibitory effects of DPP-8/9 were due to cytotoxicity. RAW264.7 cells were treated with 1G244 at concentrations ranging from 1 to 160 µM for 48 hr. In the normal group, macrophages were exposed to DMEM-only media, while the control group received RANKL+LPS. The findings revealed that 1G244 consistently reduced the RAW264.7 cell viability in a dose-dependent manner, with significant cell death observed at concentrations above 80 µM. Notably, 1G244 at concentrations up to 20 µM maintained cell viability in RANKL+LPS-treated RAW264.7 macrophages. Based on these findings, concentrations of 1G244 up to 20 µM were selected for all the study experiments (Figure 1). 

### Evaluation of DPP-8/9 and its inhibition in RAW-osteoclast cell

**Figure 2:**
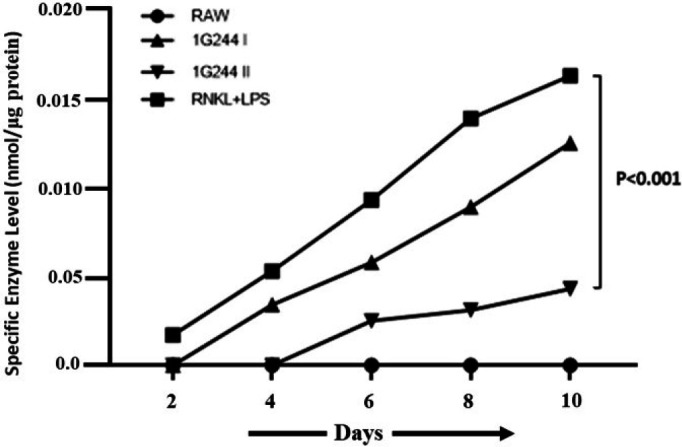
DPP-8/9 activity in RAW264.7 cell lysates on the 2^nd^, 4^th^, 6^th^, 8th, and 10^th^ day with and without DPP inhibitors. RAW264.7 cell lysate with 50 µg/ml of RANKL and 20 ng/ml of LPS was used as the negative control for the DPP-8/9 assay. All values are presented as the mean ± SEM (n=3). Statistical significance was determined by one-way ANOVA with Tukey’s multiple comparison test. #*P*<0.05 and ##*P*<0.01 and ###*P*<0.001, when compared with the control group (group I), *P<0.05, and **P<0.01, when compared vs RANKL+LPS group (group II).

RAW264.7 macrophage cell lysates treated with a RANKL+LPS solution contained 0.0153 nmol/µg of DPP-8/9 activity (Figure 2). The DPP-8/9 activity in the RANKL+LPS-treated lysates increased progressively up to the 10th day. However, lysates from the normal group treated with DMEM alone showed no DPP-8/9 activity. When these cell lysates were subsequently treated with 1G244 at concentrations of 10 µM and 20 µM, the DPP-8/9 activity was reduced to 0.0116 nmol/µg and 0.0037 nmol/µg compared to the untreated RANKL+LPS group. The RANKL+LPS treatment resulted in a 73.17% increase in enzyme activity compared to 1G244 untreated lysates. Treatment with 10 µM 1G244 reduced DPP-8/9 activity insignificantly by 17.86% to 0.0116 nmol/µg. In comparison, treatment with 20 µM 1G244 on the 10th day led to a 66.2% significant reduction in specific enzyme activity to 0.0037 nmol/µg (*P*<0.001) in contrast to the RANKL+LPS administered group (Figure 2).

### Effect of DPP-8/9 inhibition on inflammatory cytokines (TNF-α, IL-23 and IL-6)

**Figure 3:**
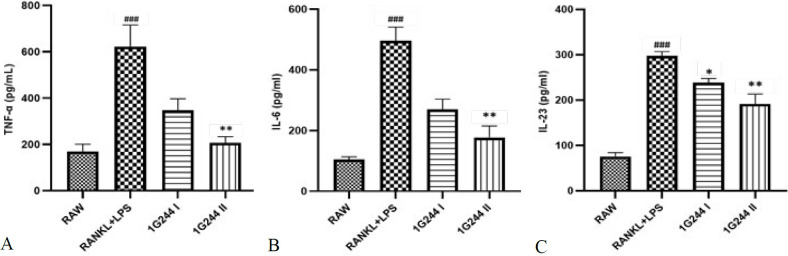
Effect of 1G244 on TNF-α (a), IL-6 (b), and IL-23 (c). RAW264.7 cells were pretreated with RANKL (50 ng/ml) for five days

Exposure of RAW264.7 cells to LPS and RANKL triggered the inflammatory response, resulting in a marked elevation in IL-6, IL-23, and TNF-α levels (*P*<0.001) (Figure 3). In contrast, untreated cells showed no significant activity of these inflammatory cytokines. The introduction of the DPP-8/9 inhibitor 1G244 (20 μM) decreased the TNF-α and IL-6 (*P*<0.01) levels significantly as compared to the RANKL+LPS treated cells (Figure 3a, b). Additionally, administering 1G244 (10 μM and 20 μM) notably decreased the levels of the M1 macrophage response marker IL-23 (*P*<0.05 and *P*<0.01) (Figure 3c). This result implicates the reduction of M1 macrophage levels along with inflammatory cytokines after the treatment with 1G244.

### Effects of selective DPP-8/9 inhibition on macrophage polarization in RAW264.7 cell

**Figure 4:**
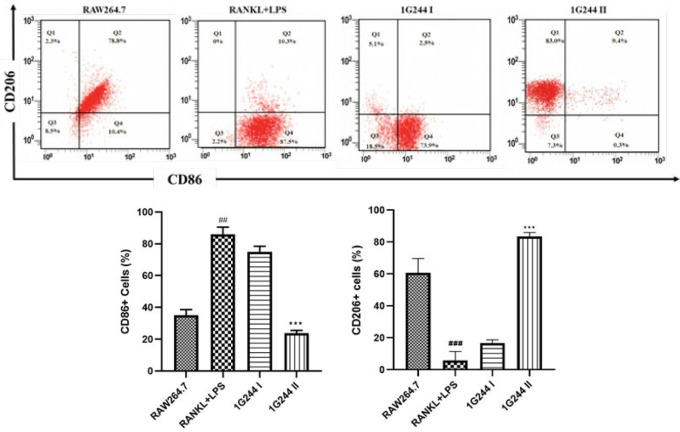
Cell surface markers (analyzed via flow cytometry) in RAW264.7 cells. Markers CD206 and CD86 in RAW264.7

To study macrophage polarization and plasticity, we utilized RAW264.7 macrophages stimulated by LPS and RANKL to represent the two opposing polarized states: M1 and M2 phenotypes. Cell polarization assay was employed to assess the expression of macrophage markers CD86 and CD206, which are strongly associated with M1 and M2 polarization, respectively. Stimulation with LPS and RANKL shifted RAW264.7 cells toward the M1 phenotype, as indicated by an increased expression of CD86 compared to non-treated cells, highlighting the balanced expression of CD86 and CD206. However, treatment with 1G244 at a concentration of 20 µM nearly abolished CD86 expression in cells pretreated with LPS and RANKL, compared to the LPS + RANKL treated group (*P*<0.001). In contrast, a lower dose of 1G244 (10 µM) did not significantly alter CD86 expression in M1-like macrophages. These results suggest that DPP-8/9 signaling is critical in the polarization of M1 macrophages triggered by LPS and RANKL in RAW264.7 cells (Figure 4). 

### Effect of selective DPP-8/9 inhibition on TRAP-positive multinucleated osteoclasts

**Figure 5:**
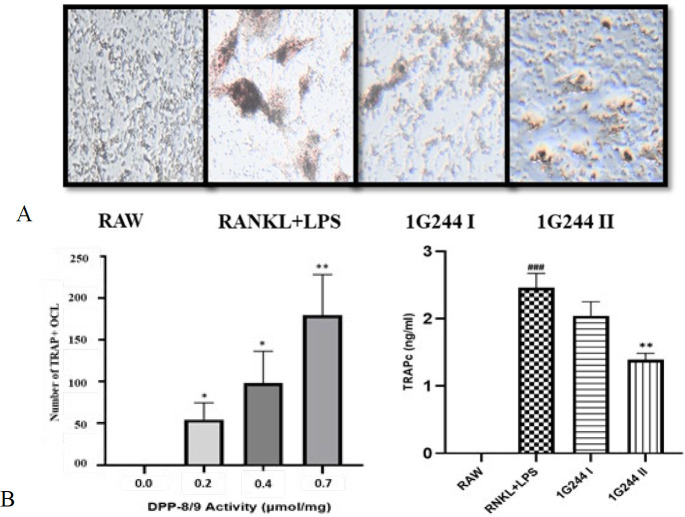
TRAPc activity in RAW264.7 cell lysates with and without 1G244 treatment

The supernatant obtained from RAW-264.7 cell pretreated with RANKL and LPS solution showed significant elevation in TRAPc level compared to the untreated controlled cells (*P*<0.001). The RANKL and LPS administered RAW-264.7 cells were treated with selective DPP-8/9 inhibitor 1G244 (20 μM) and showed a significant reduction in TRAPc level (*P*<0.01) (Figure 5C). 

During visualization of RAW264.7 macrophages in various groups, the number of TRAP^+^ osteoclasts in the RANKL+LPS group was highest (>150) with the highest DPP-8/9 activity (0.7 μmol/mg) (*P*<0.01). Whereas, the treatment with 1G244 (10 & 20 μM) decreased the number of TRAP^+^ osteoclasts (<100) as well as DPP-8/9 levels (0.4 and 0.2 μmol/mg) as compared to the RANKL+LPS pretreated cells, significantly (*P*<0.05 and *P*<0.01) (Figure 5A, B). 

### Interactions of molecular docking pose at the binding site of DPP-8/9 receptors

**Figure 6:**
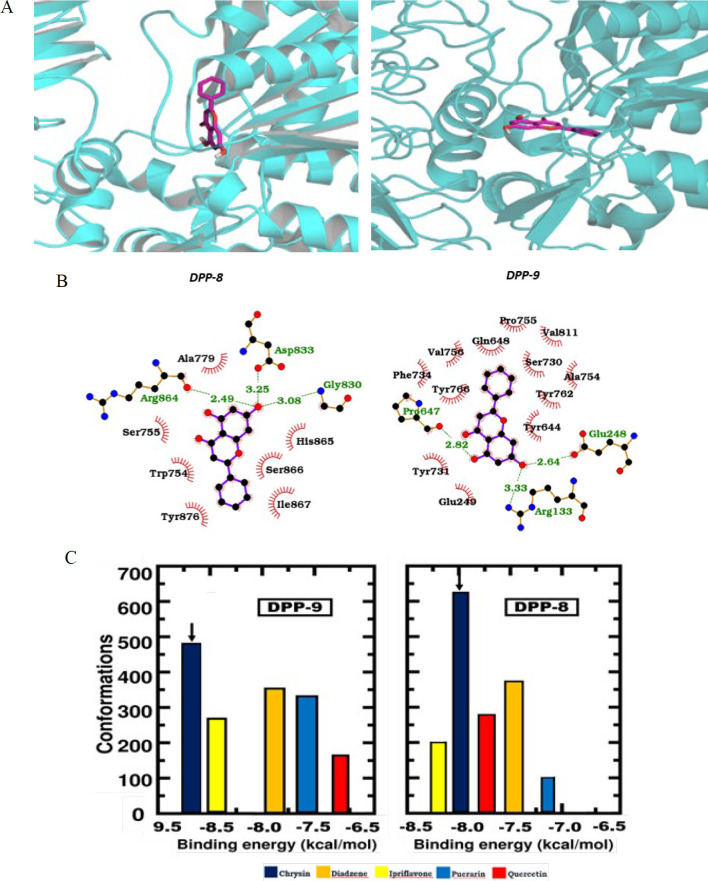
(A) Zoom-in view of the docked pose of the chrysin molecule shown in magenta color

Various ligands positioned within the binding pocket of both DPP-8 and DPP-9 receptors were generated, and the best-docked conformation compound is illustrated in Figure 6. Notably, the ligand, chrysin, with the most favorable conformation occupies distinct positions within the binding sites of both DPP-8/9 receptors. The docked complexes were ranked according to their geometric shape complementarity, emphasizing optimal fit with a large interface area and minimal steric hindrance. In this study, five compounds were shortlisted for molecular docking: ipriflavone, quercetin, diadzine, puerarin, and chrysin. These flavonoids have been extensively studied in pharmacological research, establishing a solid foundation for computational modeling and making them reliable candidates for comparison with other derivatives (41). Moreover, each compound is linked to specific therapeutic applications, such as anti-oxidant properties or bone health benefits (32). The binding affinity details of standard 1G244, chrysin, and other flavonoids with the active binding site of DPP-8/9 are presented in Table 1. The docking results revealed that chrysin (binding energy: 8.0 kcal/mol with DPP-8 and 9.9 kcal/mol with DPP-9) showed comparable binding interactions within the ligand-binding domain of both enzymes. These interactions align closely with those observed for the reference inhibitor, 1G244 (binding energy: 9.8 kcal/mol with DPP-8 and 10.0 kcal/mol with DPP-9), suggesting that chrysin may possess similar inhibitory potential against DPP-8/9 (Figure 6; Table 1).

Examining the interactions between amino acid residues at the binding site of DPP-8/9 receptors and the ligand (chrysin) revealed unconventional interlinkages involving pi interactions, hydrophobic side chains, and hydrogen bonds. For DPP-8, amino acid residues such as GLY 830, ASP 833, and ARG 864 formed a hydrogen bond with 5-OH of chrysin, while ALA 779, SER 755, TRP 754, TYR876, SER866, ILE867, and HIS 865 were connected through van der Waals interaction. Similarly, in the case of DPP-9, amino acid residues, including GLY 248, ARG 133, and PRO 647, formed a hydrogen bond with 5-OH of chrysin. Additionally, GLU 249, ALA 754, SER 730, GLN648, TYR731, and VAL 756 were bonded via van der Waals forces (6A, B).

### Western blotting

**Figure 7:**
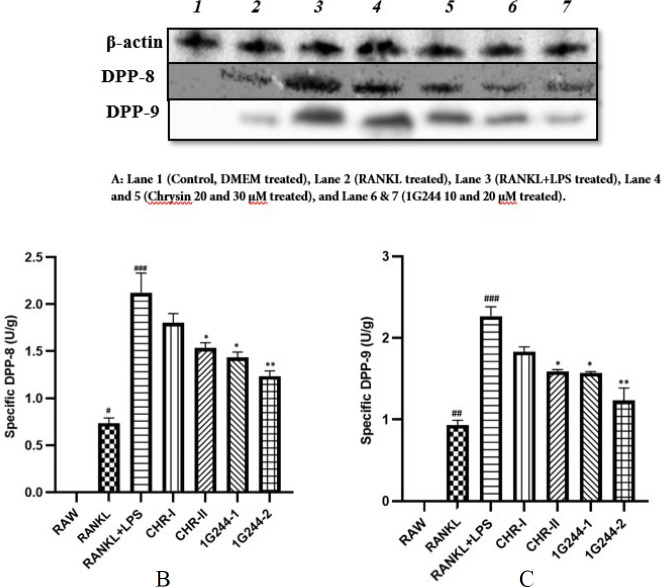
Increased protein expression levels of DPP-8 and DPP-9 in RAW264.7 cell

This analysis was conducted to assess the impact of chrysin on DPP-8/9 expression in RAW264.7 cell cultures. The cell viability assay for chrysin indicated that 20 and 30 μM concentrations of chrysin show no adverse effect on the RAW 264.7 cells, and it could significantly enhance cell proliferation in a dose-dependent manner (data not shown). Compared to the control group, RANKL and LPS treatments elevated the DPP-8/9 expression significantly (*P*<0.001). Notably, DPP-8/9 expression was also significantly higher in the cells treated with only RANKL (*P*<0.05 and *P*<0.01). When evaluating the impact of chrysin at concentrations of 20 and 30 μM on RANKL+LPS-induced osteoclasts, treatment with 30 μM chrysin reduced DPP-8/9 expression compared to the untreated groups (*P*<0.05). Additionally, DPP-8/9 expression was significantly reduced after the 1G244 treatment (10 and 20 μM) as compared to the untreated groups (*P*<0.01). The reduction in DPP-8/9 expression was more pronounced in the 1G244 (20 μM) treated cells compared to the chrysin-treated group (30 μM) (*P*<0.01) (Figure 7).

## Discussion

Chronic inflammation significantly threatens bone health by inducing osteoclastogenesis (12). This complex process depends on the inflammatory cytokines and the precise recruitment and fusion of inflammatory macrophages, which act as osteoclast progenitors (42–44). Existing drugs targeting osteoclasts have shown limited effectiveness in inflammatory habitats, highlighting the necessity for alternative therapeutic approaches to address inflammation-related bone loss (18, 45). Previous studies have noted increased expression of the DPP-8/9 during inflammation, with the inhibition of these enzymes demonstrating potential in mitigating inflammatory macrophage activation (26,27). Here, we examined the DPP-8/9 role in inflammation-related osteoclastogenesis, specifically using RAW-264.7 murine macrophages to analyze its impact.

The RAW 264.7 model has been instrumental in osteoclastogenesis research for over two decades, owing to its availability and homogeneous population of primary osteoclasts (36). This cell line is highly versatile, containing a nearly equal mix of inflammatory (M1-type) and non-inflammatory (M2-type) types of macrophages, which can be polarized into either M1-type or M2-type cells (46). However, its significance extends beyond its accessibility and versatility, as it mimics human skeletal responses, particularly in inflammatory scenarios (47). This model has become essential for studying inflammation-induced osteoclast formation and investigating the involvement of DPP-8/9 enzymes. To elucidate the role of DPP-8/9 in this process, we employed its selective inhibitor 1G244 (Figure 1) (22, 33). Despite initial concerns about its cytotoxicity, lower concentrations of 1G244 (10 and 20 µM) effectively penetrate cell membranes and inhibit DPP-8/9 activity without inducing significant cell death (48, 49). It helped us to maintain accurate concentration in the media and mitigate potential toxic effect reactions on the cells (Figure 2).

LPS significantly enhances the secretion of inflammatory mediators, creating an environment that promotes the polarization of M1 macrophages within the RANKL-primed cell lineage, thereby further stimulating osteoclastogenesis (46,50). The process triggers the release of cytokines (TNF-α, IL-23, and IL-6), facilitating the formation of large TRAP-positive osteoclasts (47). However, administration of 1G244 attenuated this elevation, resulting in decreased levels of TNF-α, IL-23, and IL-23, which plays a role in immune regulation and bone loss in chronic inflammatory diseases like osteoporosis (39). The reduction in IL-23 levels observed in the 1G244 treatment group holds significant therapeutic promise, as it influences the osteoclast formation in an inflammatory environment due to its direct impact on the precursors of osteoclast (14) (Figure 3). It is predominantly synthesized by innate immune cells, macrophages and dendritic cells (DC), and involved in inflammatory and autoimmune disorders along with other cytokines (39). Genetically modified IL-23 gene-deactivated mice were sheltered from any skeletal destruction in the collagen-induced arthritis model (14). This observation implies that the administration of DPP-8/9 inhibitor significantly reduces inflammatory cytokines in the treatment groups.

When exposed to proinflammatory agents LPS and RANKL, RAW-macrophages experience significant proliferation along with elevated levels of DPP-8/9 enzymes (27) (Figure 2). Macrophage polarization is generally divided into two categories: classically activated M1-macrophages and alternatively activated M2-macrophages (46). When proinflammatory signals like LPS activate macrophages, they undergo a metabolic shift from oxidative phosphorylation to aerobic glycolysis, a phenomenon referred to as the Warburg effect (51). The M1 and M2 macrophage phenotypes differ in their cytokine and chemokine secretion and exhibit distinct morphological characteristics (11). M1-type contributes to elevated bone loss through various pathways, including inflammatory cytokines release. Additionally, they can potentially transform into multinucleated osteoclasts (10). CD86 is a widely recognized marker for M1 macrophage polarization, while CD206 is typically used to identify M2 macrophages. LPS stimulates RAW264.7 macrophages to produce large amounts of NO, ROS, and proinflammatory cytokines that might shift the non-specific expression of M2 macrophages into inflammatory M1 macrophages (50). Various studies propose that DPP-8/9 may regulate the transition between these two polarization states (24, 26, 27). The LPS + RANKL administration elevated the expression of CD86 markers of M1-type macrophages. However, treatment with 1G244 significantly decreased CD86 expression while increasing CD206 expression (Figure 4). These findings suggest that overexpression of DPP-8/9 found in M1 macrophage polarized in an inflammatory environment and 1G244 could potentially reduce the inflammatory response in RAW264.7 macrophages by preventing their conversion into M1 macrophages (Figure 4).

The RAW264.7 cells’ differentiation into TRAP^+ ^osteoclasts is a well-regulated process triggered by RANKL stimulation (47). Any interference in this pathway can disrupt osteoclast formation, affecting the outcomes of related experiments (47, 52). TRAP activity is a key marker for osteoclast function, playing a critical role in maintaining bone resorption rates (52, 53). Accurate assessment of osteoclast abundance can be achieved through TRAP staining and quantification (47, 53). In our study, treatment with the DPP-8/9 inhibitor 1G244 significantly decreased the TRAP^+^ cell number compared to the untreated, toxic group (*P*<0.01) (Figure 5a,c). By the 10th day, DPP-8/9 activity was measured, showing no activity in the DMEM-treated control group. In contrast, the RANKL+LPS group exhibited the highest activity level (0.7 μmol/mg) (Figure 5b). The inflammatory treatment (RANKL and LPS), intended to induce the transformation of RAW-macrophages into osteoclasts, resulted in elevated DPP-8/9 levels in these cells, particularly in mature osteoclasts (Figure 5b). This suggests that DPP-8/9 may be up-regulated during the formation of osteoclasts from M1 macrophages. Although the exact role of DPP-8/9 in inflammatory osteoclastogenesis is not fully understood, our results propose that M1-type macrophages take part in this process, especially in an inflammatory setting. Treatment with 1G244 significantly reduced osteoclast formation, indicating that aiming DPP-8/9 could be a potential approach against inflammatory bone loss (24, 26). These results highlight the need for further research into these proteins as potential therapeutic targets.

Clinical inhibitors that target osteoclast function often fail to treat osteolytic conditions effectively, and they can lead to significant negative side effects (54). The adverse effects associated with 1G244 have been particularly challenging to use *in vivo *studies. Interestingly, previous studies have reported that these side effects are not due to DPP-8/9 inhibition itself but rather other factors related to 1G244 (33). This underscores the urgent need for inhibitors specifically designed to target DPP-8/9, which could offer new avenues for treating inflammatory bone loss. Scientists are now synthesizing selective and safer analogies for these proteases with reference to the 1G244 structure (27,49). Here, we employed molecular docking to assess the binding affinities of various flavonoids with DPP-8 and DPP-9 (Figure 6). Our results revealed that chrysin exhibited notable binding affinities with DPP-8 (-37.65 KJ/mol) and DPP-9 (-41.4 KJ/mol), outperforming other flavonoids tested (Figure 6c; Table 1). Hydrogen bonding between chrysin’s hydroxyl groups and DPP-8/9 proteins likely enhances their interactions (Figure 6b). Chrysin is generally recognized as a safe compound (GRAS). Previous studies have established a high safety profile for chrysin, with an LD50 of 4350 mg/kg in rats (55). Human trials have also confirmed that oral doses between 200 to 625 mg are safe and non-toxic (56). Administration of chrysin at concentrations of 20 and 30 μM did not affect cell viability, allowing for uninterrupted study progression. These results highlight chrysin as a promising molecule for further exploration and validation of its potential therapeutic effects in reducing inflammation-triggered osteoclastogenesis.

Further study supported by a comprehensive investigation of RAW-osteoclasts through immunoblotting. DPP-8/9 expression was significantly elevated in the RANKL+LPS-treated cell group compared to the DMEM-treated control group cells (*P*<0.01) (Figure 7). The pronounced elevation in the expression of DPP-8/9 observed in the toxic group aligns with earlier findings, supporting the hypothesis that elevation in DPP-8/9 expression occurs in RAW264.7 cells under inflammatory conditions (20). To assess the RANKL effect on the polarization of RAW264.7 cells and the expression of DPP-8/9, RANKL was used on its own. The resulting protein bands were weak, reflecting a limited level of expression (Figure 7). These findings imply that RANKL by itself is insufficient, and its combination with LPS triggers a robust inflammatory response needed for M1 macrophage polarization and the formation of osteoclasts. While chrysin inhibited DPP-8/9 proteins at higher doses, its inhibitory activity was less pronounced than that of 1G244. 

In osteoimmunology, inflammatory macrophages are key precursors to osteoclasts, playing a pivotal role in osteoclastogenesis driven by chronic inflammation and excessive cytokine production (7, 19). In our study, elevated TRAP^+^ levels highlight this process (6, 15) (Figure 5). DPP-8/9 levels were significantly higher in RAW cells administered with LPS + RANKL, a trend that was persistent in untreated cells (Figures 2, 4, and 5). However, inhibiting DPP-8/9 using 1G244 resulted in markedly suppressing inflammatory macrophages, evidenced by decreased CD68 markers, lower cytokine levels, and fewer osteoclasts (Figures 2, 3, and 4). These results highlight a multiple-fold increase in DPP-8/9 levels in the cells with a high number of macrophages after inflammatory treatment (24, 26, 27). Additionally, inhibition of DPP-8/9 has effectively reduced inflammatory macrophage activation, suppressing cytokine levels (26). These findings confirm DPP-8/9 involvement in inflammation and suggest that targeting it could offer a promising strategy for treating inflammatory bone disorders such as autoimmune rheumatoid arthritis (33). These results reinforce previous findings and support our hypothesis that DPP-8/9 levels are crucial in inflammatory osteoclastogenesis, as demonstrated in RAW264.7 cells (33). Moreover, chrysin, a flavonoid, exhibited potential anti-DPP-8/9 activity, highlighting its therapeutic promise (Figure 7). Further research on its inhibitory effects could be crucial for developing treatments for inflammatory osteoclastogenesis. Despite various limitations, such as the lack of essential tools and unknown substrates affecting chrysin’s mechanism on DPP-8/9, our results align with previous research, emphasizing the enzyme’s role in inflammation. This study provides preliminary preclinical evidence that DPP-8/9 could be a therapeutic target for inflammatory conditions, highlighting the need for further investigation.

Cells were administered 1G244 (1-160 μM) for 48 hr. The normal group was administered only DMEM media, and the control group was administered inflammatory agents RANKL+LPS solution. Graphs depict mean ± SEM. n = 12 wells per group. *P<0.05, **P<0.01 vs Control.

On the seventh day, LPS (20 ng/ml) was added for 8 hr, followed by the administration of 1G244 at concentrations of 10 and 20 µM. All values are presented as the mean ± SEM (n=3). Statistical significance was determined by one-way ANOVA with Tukey’s multiple comparison tests; #*P*<0.05, ##*P*<0.01, ###*P*<0.001 compared to the control group (untreated RAW264.7 cells); *P<0.05, **P<0.01 compared to the RANKL+LPS group.

cells before and after administering inflammatory agents RANKL+LPS. The effect of different doses (10 and 20 µM) of 1G244 was also evaluated in the experiment. All values are presented as the mean ± SEM (n=3). Statistical significance was determined by one-way ANOVA with Tukey’s multiple comparison test. ##*P*<0.01, ###*P*<0.001 compared to the control group (DMEM treated cells); ***P<0.001 compared to the RANKL+LPS group.

A) RAW264.7 macrophages treated with and without RANKL (50 ng/ml) and LPS (20 ng/ml) followed by 1G244 treatment for the measurement of TRAP and DPP-8/9 activity. B) Relationship between DPP-8/9 and TRAP-positive osteoclasts at different days of TRAPc activity. C) On the 10^th^ day, cells were lysed to release the dye, and the absorbance was measured for TRAP activity. All values are presented as the mean ± SEM (n=3). Statistical significance was determined by one-way ANOVA with Tukey’s multiple comparison test. ###*P*<0.001 when compared with RAW group, *P<0.05, and **P<0.01, when compared with RANKL+LPS group.

Chrysin binds to DPP-8 and DPP-9 with a binding energy sufficient to produce the inhibition. (B) Residue level interaction between chrysin and DPP-8 and DPP-9. The substrate and protein residues involved in H-bonds are shown in ball and stick form. All the other residues of proteins involved in hydrophobic interactions are represented in red spikes (c). Number of clusters formed during docking is shown as a histogram. Number of conformations falling in a particular cluster and the average binding energy of that cluster are shown in the Y and X axes, respectively. The RMSD cut-off of 2 Å is taken for clustering. The cluster chosen as the final pose is highlighted with a black arrow.

**Table 1:** Mean binding energy (Kj/mol) of 1G244 and various flavonoid compounds with DPP-8 and DPP-9 proteins using molecular docking

Mean **binding energy (kJ/mol)**		
**Compound**	DPP-8	DPP-9
**1G244**	41.42	42.04
**Chrysin**	37.65	41.4
**Diadazine**	32.46	32.80
**Ipriflavone**	33.97	37.44
**Puerarin**	29.91	32.38
**Quercetin**	31.71	29.41

(A) Total proteins were extracted from RAW264.7 cell (normal), RANKL, and LPS-treated cells with and without drug treatment. (B & C) Quantification of protein DPP-8 and DPP-9 expression in RAW264.7 cells treated with RANKL and LPS and their treatment with chrysin and 1G244 at different doses. All values are presented as the mean ± SEM (n=3). Statistical significance was determined by one-way ANOVA with Tukey’s multiple comparison test. #*P*<0.05, ##*P*<0.01, and ###*P*<0.001 when compared with control group, *P<0.05, and **P<0.01, when compared vs VCD groups.

## Conclusion

This research demonstrates the essential role of DPP-8/9 in driving osteoclast differentiation during inflammatory osteoclastogenesis. We observed a significant up-regulation of DPP-8/9 expression in RAW macrophages following stimulation with inflammatory mediators. Conversely, inhibition of these proteases using 1G244 resulted in decreased levels of inflammatory cytokines, M1 macrophage markers, and TRAP-positive osteoclasts, highlighting the impact of DPP-8/9 in inflammation and osteoclastogenesis. 

Given the challenge of developing selective inhibitors for DPP-8 and DPP-9 owing to their structural similarity, our identification of chrysin as a promising DPP-8/9 inhibitor molecule presents a novel opportunity for designing more targeted therapeutic strategies. Although the precise mechanism of chrysin’s action remains a limitation, this study positions DPP-8/9 as a promising therapeutic target. It suggests chrysin as a potent lead compound for managing inflammatory bone loss, thus paving the way for future translational research and drug development. 

## Data Availability

The datasets used or analyzed during the current study are available from the corresponding author upon reasonable request.

## Declaration

We have not used any AI tools or technologies to prepare this manuscript.

## References

[1] Weitzmann MN (2017). Bone and the immune system. Physiol Behav.

[2] Tsukasaki M, Takayanagi H (2019). Osteoimmunology: Evolving concepts in bone-immune interactions in health and disease. Nat Rev Immunol.

[3] Okamoto K, Nakashima T, Shinohara M, Negishi-Koga T, Komatsu N, Terashima A (2017). Osteoimmunology: The conceptual framework unifying the immune and skeletal systems. Physiol Rev.

[4] Salari N, Ghasemi H, Mohammadi L, Behzadi M hasan, Rabieenia E, Shohaimi S (2021). The global prevalence of osteoporosis in the world: A comprehensive systematic review and meta-analysis. J Orthop Surg Res.

[5] The Economist Intelligence Unit (2017). Demystifying ageing lifting the burden of fragility fractures and osteoporosis in Asia-Pacific.

[6] Srivastava RK, Sapra L (2022). The Rising Era of “Immunoporosis”: Role of Immune System in the Pathophysiology of Osteoporosis. J Inflamm Res.

[7] Dar HY, Azam Z, Anupam R, Mondal RK, Srivastava RK (2018). Osteoimmunology: The nexus between bone and immune system. Front Biosci.

[8] Khoury MI (2024). Osteoporosis and inflammation: Cause to effect or comorbidity?. Int J Rheum Dis.

[9] Pietschmann P, Mechtcheriakova D, Meshcheryakova A, Föger-Samwald U, Ellinger I (2016). Immunology of osteoporosis: A mini-review. Gerontology.

[10] Sun Y, Li J, Xie X, Gu F, Sui Z, Zhang K (2021). Macrophage-osteoclast associations: Origin, polarization, and subgroups. front immunol.

[11] Muñoz J, Akhavan NS, Mullins AP, Arjmandi BH (2020). Macrophage polarization and osteoporosis: A review. Nutrients.

[12] Michalski MN, McCauley LK (2017). Macrophages and skeletal health. Pharmacol Ther.

[13] Madel MB, Ibáñez L, Wakkach A, De Vries TJ, Teti A, Apparailly F (2019). Immune function and diversity of osteoclasts in normal and pathological conditions. Front Immunol.

[14] Chen SY, Tsai TC, Li YT, Ding YC, Wang CT, Hsieh JL (2022). Interleukin-23 mediates osteoclastogenesis in collagen-induced arthritis by modulating microRNA-223. Int J Mol Sci.

[15] Allard-Chamard H, Carrier N, Dufort P, Durand M, De Brum-Fernandes AJ, Boire G (2020). Osteoclasts and their circulating precursors in rheumatoid arthritis: Relationships with disease activity and bone erosions. Bone Reports.

[16] Saxena Y, Routh S, Mukhopadhaya A (2021). Immunoporosis: Role of innate immune cells in osteoporosis. Front Immunol.

[17] Verron E, Bouler JM (2014). Is bisphosphonate therapy compromised by the emergence of adverse bone disorders?. Drug Discov Today.

[18] Peris P, Monegal A, Guañabens N (2021). Bisphosphonates in inflammatory rheumatic diseases. Bone.

[19] Ponzetti M, Rucci N (2019). Updates on osteoimmunology: What’s new on the cross-talk between bone and immune system. Front Endocrinol (Lausanne).

[20] Cui C, Tian X, Wei L, Wang Y, Wang K, Fu R (2022). New insights into the role of dipeptidyl peptidase 8 and dipeptidyl peptidase 9 and their inhibitors. Front Pharmacol.

[21] Oikonomopoulou K, Diamandis EP, Hollenberg MD, Chandran V (2018). Proteinases and their receptors in inflammatory arthritis: An overview. Nat Rev Rheumatol.

[22] Wu JJ, Tang HK, Yeh TK, Chen CM, Shy HS, Chu YR (2009). Biochemistry, pharmacokinetics, and toxicology of a potent and selective DPP8/9 inhibitor. Biochem Pharmacol.

[23] Kalhotra P, Chittepu VCSR, Osorio-Revilla G, Gallardo-Velázquez T (2018). Structure–activity relationship and molecular docking of natural product library reveal chrysin as a novel dipeptidyl peptidase-4 (DPP-4) inhibitor: An integrated in silico and in vitro study. Molecules.

[24] Matheeussen V, Waumans Y, Martinet W, Van Goethem S, Van Der Veken P, Scharpé S (2013). Dipeptidyl peptidases in atherosclerosis: Expression and role in macrophage differentiation, activation and apoptosis. Basic Res Cardiol.

[25] Waumans Y, Baerts L, Kehoe K, Lambeir AM, De Meester I (2015). The dipeptidyl peptidase family, prolyl oligopeptidase and prolyl carboxypeptidase in the immune system and inflammatory disease, including atherosclerosis. Front Immunol.

[26] Waumans Y, Vliegen G, Maes L, Rombouts M, Declerck K, Van Der Veken P (2016). The dipeptidyl peptidases 4, 8, and 9 in mouse monocytes and macrophages: DPP8/9 inhibition attenuates M1 macrophage activation in mice. Inflammation.

[27] Suski M, Wiśniewska A, Kuś K, Kiepura A, Stachowicz A, Stachyra K (2020). Decrease of the pro-inflammatory M1-like response by inhibition of dipeptidyl peptidases 8/9 in THP-1 macrophages - quantitative proteomics of the proteome and secretome. Mol Immunol.

[28] Buljevic S, Detel D, Pugel EP, Varljen J (2018). The effect of CD26-deficiency on dipeptidyl peptidase 8 and 9 expression profiles in a mouse model of Crohn’s disease. J Cell Biochem..

[29] Chowdhury S, Chen Y, Yao TW, Ajami K, Wang XM, Popov Y (2013). Regulation of dipeptidyl peptidase 8 and 9 expression in activated lymphocytes and injured liver. World J Gastroenterol.

[30] Wilson CH, Abbott CA (2012). Expression profiling of dipeptidyl peptidase 8 and 9 in breast and ovarian carcinoma cell lines. Int J Oncol.

[31] Torrecillas-Baena B, Camacho-Cardenosa M, Quesada-Gómez JM, Moreno-Moreno P, Dorado G, Gálvez-Moreno MÁ (2023). Non-specific inhibition of dipeptidyl peptidases 8/9 by dipeptidyl peptidase 4 inhibitors negatively affects mesenchymal stem cell differentiation. J Clin Med.

[32] Nile SH, Keum YS, Nile AS, Jalde SS, Patel RV (2018). Anti-oxidant, anti-inflammatory, and enzyme inhibitory activity of natural plant flavonoids and their synthesized derivatives. J Biochem Mol Toxicol.

[33] Ahmad SS, Ahmed F, Alam MM, Ahmad S, Khan MA (2024). Unravelling the role of dipeptidyl peptidases-8/9 (DPP-8/9) in inflammatory osteoporosis: a comprehensive study investigating chrysin as a potential anti-osteoporotic agent. J Pharm Pharmacol.

[34] Chang Y, Hawkins BA, Du JJ, Groundwater PW, Hibbs DE, Lai F (2023). A guide to in silico drug design. Pharmaceutics.

[35] Bhuvaneshwari S, Sankaranarayanan K (2019). Identification of potential CRAC channel inhibitors: Pharmacophore mapping, 3D-QSAR modelling, and molecular docking approach. SAR QSAR Environ Res.

[36] Lampiasi N, Russo R, Kireev I, Strelkova O, Zhironkina O, Zito F (2021). Osteoclasts differentiation from murine RAW 264 7 cells stimulated by RANKL: Timing and behavior. Biology (Basel).

[37] Ghasemi M, Turnbull T, Sebastian S, Kempson I (2021). The mtt assay: Utility, limitations, pitfalls, and interpretation in bulk and single-Cell analysis. Int J Mol Sci.

[38] Smith TD, Tse MJ, Read EL, Liu WF (2016). Regulation of macrophage polarization and plasticity by complex activation signals. Integr Biol.

[39] Shukla P, Mansoori MN, Singh D (2018). Efficacy of anti-IL-23 monotherapy versus combination therapy with anti-IL-17 in estrogen deficiency induced bone loss conditions. Bone.

[40] Kitaura H, Marahleh A, Ohori F, Noguchi T, Shen WR, Qi J (2020). Osteocyte-related cytokines regulate osteoclast formation and bone resorption. Int J Mol Sci.

[41] Ysrafil Y, Sapiun Z, Slamet NS, Mohamad F, Hartati H, Damiti SA (2023). Anti-inflammatory activities of flavonoid derivates. ADMET DMPK..

[42] Ahmad SS, Ahmed F, Ali R, Ghoneim MM, Alshehri S, Najmi AK (2022). Immunology of osteoporosis: Relevance of inflammatory targets for the development of novel interventions. Immunotherapy.

[43] Toker H, Ozdemir H, Balci Yuce H, Goze F (2016). The effect of boron on alveolar bone loss in osteoporotic rats. J Dent Sci.

[44] Xu F, Teitelbaum SL (2013). Osteoclasts: New Insights. Bone Res.

[45] Lu L, Lu L, Zhang J, Li J (2020). Potential risks of rare serious adverse effects related to long-term use of bisphosphonates: An overview of systematic reviews. J Clin Pharm Ther.

[46] Hwang J, Zheng M, Wiraja C, Cui M, Yang L, Xu C (2020). Reprogramming of macrophages with macrophage cell membrane-derived nanoghosts. Nanoscale Adv.

[47] Kong L, Smith W, Hao D (2019). Overview of RAW264 7 for osteoclastogensis study: Phenotype and stimuli. J Cell Mol Med.

[48] Han R, Wang X, Bachovchin W, Zukowska Z, Osborn JW (2015). Inhibition of dipeptidyl peptidase 8/9 impairs preadipocyte differentiation. Sci Rep.

[49] Kikuchi S, Wada A, Kamihara Y, Okazaki K, Jawaid P, Rehman MU (2023). DPP8 selective inhibitor tominostat as a novel and broad-spectrum anticancer agent against hematological malignancies. Cells.

[50] Khabipov A, Käding A, Liedtke KR, Freund E, Partecke LI, Bekeschus S (2019). RAW 264 7 macrophage polarization by pancreatic cancer cells - A model for studying tumour-promoting macrophages. Anticancer Res.

[51] Kelly B, O’Neill LAJ (2015). Metabolic reprogramming in macrophages and dendritic cells in innate immunity. Cell Res.

[52] Cheng Y, Liu H, Li J, Ma Y, Song C, Wang Y (2022). Evaluation of culture conditions for osteoclastogenesis in RAW264 7 cells. PLoS One.

[53] Deng C, Zhang Q, He P, Zhou B, He K, Sun X (2021). Targeted apoptosis of macrophages and osteoclasts in arthritic joints is effective against advanced inflammatory arthritis. Nat Commun.

[54] Wu Z, Li C, Chen Y, Liu Q, Li N, He X (2022). Chrysin protects against titanium particle-induced osteolysis by attenuating osteoclast formation and function by inhibiting NF-κB and MAPK signaling. Front Pharmacol.

[55] Yao W, Cheng J, Kandhare AD, Mukherjee-Kandhare AA, Bodhankar SL, Lu G (2021). Toxicological evaluation of a flavonoid, chrysin: Morphological, behavioral, biochemical and histopathological assessments in rats. Drug Chem Toxicol.

[56] Walle T, Otake Y, Brubaker JA, Walle UK, Halushka PV (2001). Disposition and metabolism of the flavonoid chrysin in normal volunteers. Br J Clin Pharmacol.

